# Translocation of heme oxygenase-1 contributes to imatinib resistance in chronic myelogenous leukemia

**DOI:** 10.18632/oncotarget.18684

**Published:** 2017-06-27

**Authors:** Bianca Schaefer, Soenke Behrends

**Affiliations:** ^1^ Department of Pharmacology, Toxicology and Clinical Pharmacy, University of Braunschweig - Institute of Technology, Braunschweig, 38106, Germany

**Keywords:** heme oxygenase-1, nuclear translocation, drug resistance, chronic myelogenous leukemia, imatinib

## Abstract

Heme oxygenase-1 (HO-1) degrades heme to bilirubin. In addition, it is upregulated in malignant disease and has been described as an important factor for cancer prognosis and therapy. Under physiological conditions HO-1 is anchored to the endoplasmic reticulum (ER). Under stress conditions HO-1 can be cleaved and subsequently translocates to the cytosol and nucleus.

In this study we systematically investigated the influence of HO-1's catabolic activity and subcellular localization on resistance against the tyrosine kinase inhibitor imatinib in leukemia cells by confocal laser scanning microscopy, hemoglobin synthesis experiments and cell viability assays. We created two types of monoclonal K562 cell lines stably transfected with GFP-tagged HO-1: cell lines expressing ER anchored HO-1 or anchorless HO-1. Since translocation of HO-1 disrupts the association with cytochrome P450 reductase, heme degrading activity was higher for ER anchored versus anchorless HO-1. Cell viability tests with increasing concentrations of imatinib showed IC50-values for all six cell lines with ER localized HO-1 that were similar to control cells. However, out of the seven cell lines with anchorless HO-1, two showed a statistically significant increase in the imatinib IC50 (19.76 μM and 12.35 μM versus 2.35 – 7.57 μM of sensitive cell lines) corresponding to plasma concentrations outside the therapeutic range.

We conclude that the presence of translocated HO-1 in the cytosol and nucleus supports imatinib resistance while it is not sufficient to cause imatinib resistance in every cell line. In contrast, an increase in ER anchored HO-1 with high heme degrading activity does not contribute to imatinib resistance.

## INTRODUCTION

Heme oxygenase-1 (HO-1), an inducible heme degrading enzyme important for iron hemostasis and oxidative stress response, emerges as a novel target of cancer therapy [1]. HO-1 is upregulated in prostate cancer [2, 3], pancreas carcinoma [4], myeloid leukemia [5, 6] and lymphoblastic leukemia [7]. Under normal conditions, HO-1 is carboxy-terminally anchored to the endoplasmic reticulum (ER), but under stress conditions, for example under hypoxia, it is cleaved and translocates to the cytosol and nucleus [8, 9]. Translocation disrupts the association of HO-1 with the electron donating enzyme cytochrome P450 reductase (CPR) [8, 9]. This reduces heme degrading catalytic activity to very low levels [9]. Recently, HO-1 translocation was found to be mediated by signal peptide peptidase (SPP) [10, 11]. High levels of translocated HO-1 were observed in cell lines with high SPP expression [3, 11, 12]. In head and neck squamous cell carcinoma [12] and multiple myeloma [13] nuclear HO-1 seems to play a role in malignant progression or drug resistance. Translocation of HO-1 to the cytosol and nucleus has been explicitly linked to imatinib resistance in the chronic myelogenous leukemia cell line K562 [14]. To test whether imatinib resistance can be induced by overexpression of HO-1, we used this cell line as it is amenable to genetic manipulation and pharmacological testing.

Imatinib is a competitive inhibitor of the oncogenic tyrosine kinase BCR-ABL that causes chronic myelogenous leukemia (CML) [15–17]. Mutations in the BCR-ABL kinase domain can confer resistance to imatinib [18–21]. Beyond these classic cases of imatinib resistance, sensitivity to imatinib has been suggested to be influenced by HO-1, an enzyme that seems to have no connection to imatinib signaling at first sight. However, the observation that heme oxygenase is upregulated in myeloid leukemia [5, 6] and is induced by imatinib treatment [5], sparked interest. Further experiments showed that inhibition of HO-1's catalytic activity with zinc protoporphyrine (ZnPP) could recover imatinib sensitivity in formerly resistant cells [6, 22]. Surprisingly, a recent study indicates that imatinib resistance is mediated by nuclear HO-1 independent of its catalytic activity [14]. Thus, while all studies agree on a role of HO-1 in tumor progression and imatinib resistance, it is unclear whether this effect is mediated by the catalytic activity of HO-1 or its subcellular localization or a combination of both.

In the present study, we systematically investigated HO-1 mediated imatinib resistance in leukemia cells to analyze the relevance of HO-1's catalytic activity and subcellular localization for the development of drug resistance. We created two different types of monoclonal stable K562 cell lines overexpressing GFP-tagged HO-1:cells with full length ER resident HO-1 and cells with an anchorless HO-1 mutant that localizes to the cytosol and nucleus representing a model for translocated HO-1. We found that overexpression of ER resident HO-1 alone does not cause imatinib resistance. However, two out of seven cell lines expressing anchorless, translocated HO-1 showed imatinib resistance. We conclude that translocated HO-1 in the cytosol and nucleus seems to foster drug resistance while overexpression of ER resident HO-1 does not seem to have an influence on imatinib resistance.

## RESULTS

### Generation of cell models for ER anchored HO-1 and anchorless HO-1

To analyze the role of HO-1 in imatinib resistance, we created monoclonal stable K562 cell lines expressing GFP-HO-1 or the anchorless construct GFP-HO-1-ΔC266. We additionally generated a monoclonal stable K562 cell line expressing GFP only as a control. The expression of GFP or the different HO-1 variants was confirmed by Western blot with specific anti-GFP and anti-HO-1 antibodies. In addition, subcellular localization was analyzed using live cell imaging.

**Figure 1:**
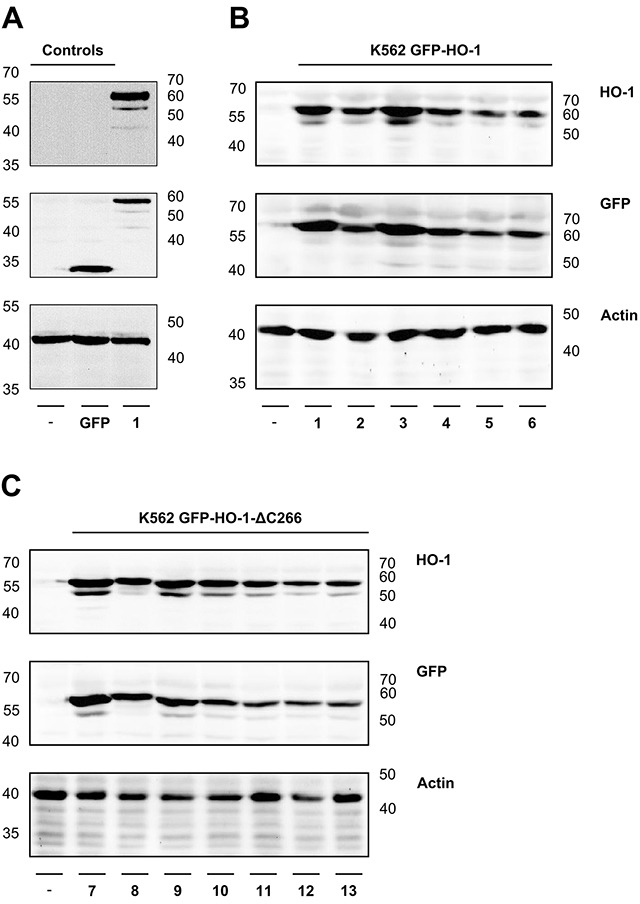
Analysis of protein expression of the generated monoclonal stable K562 cell lines by Western blot The correct protein expression of the newly generated monoclonal stable K562 cell lines was analyzed by SDS-PAGE of cell extracts, followed by Western blot, detected with specific GFP and HO-1 antibodies. Actin was detected as loading control. **(A)** Controls-: Untransfected K562. GFP: Stable K562 cell line expressing GFP. **(B)** ER resident K562 GFP-HO-1 1-6. **(C)** Anchorless variant K562 GFP-HO-1-ΔC266 7-13. Left and right: ladder [kDa].

As expected, untransfected K562 cells did not show any signal at around 60 kDa, neither with GFP antibody nor with HO-1 antibody. The K562 GFP cell line showed a signal detected with GFP antibody at around 30 kDa, but no signal for detection with HO-1 antibody (Figure 1A). All the GFP-fused HO-1 variants could be detected with GFP and HO-1 antibodies at a size of around 60 kDa (Figure 1B and 1C). Actin, which was used as loading control, was clearly detectable at around 40 kDa in all cell extracts (Figure 1). Some samples show a second band below the GFP-fused HO-1 construct. Such double bands have also been observed for HO-1 (but not HO-2) after expression in the baculovirus / Sf9 expression system [9]. It is unlikely that the lower band represents a carboxy-terminal degradation product of HO-1 as described by Yoshida et al. [23], because the carboxy-terminally deleted GFP-HO-1-ΔC266 shows a similar second band (Figure 1C and [9]). It is rather likely that the lower band represents a different post-translationally modified version of HO-1.

**Figure 2:**
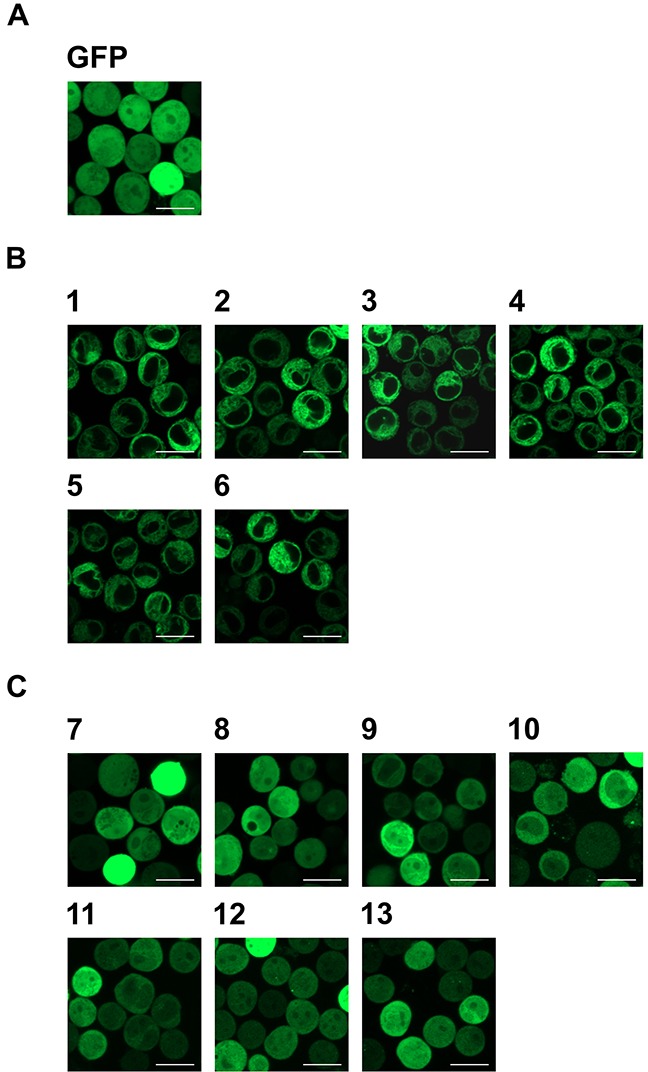
Analysis of the subcellular localization of GFP, GFP-HO-1 and GFP-HO-1-ΔC266 in the positively characterized stable K562 cell lines by confocal laser scanning microscopy **(A)** In the monoclonal stable K562 cell line expressing GFP, the protein is distributed in cytosol and nucleus. **(B)** In the monoclonal stable K562 cell lines 1-6 GFP-HO-1 is localized at the ER membrane. The nucleus is free from GFP-HO-1. **(C)** In the monoclonal stable K562 cell lines 7-13 GFP-HO-1-ΔC266 is distributed in cytosol and nucleus. Figure shows representative data from three pictures per sample measured on a CLSM at 37°C. Bar represents 20 μm.

In addition, the subcellular localization of GFP and the GFP-fused HO-1 constructs was analyzed by confocal laser scanning microscopy (Figure 2). As expected, GFP is distributed in nucleus and cytosol (Figure 2A). Monoclonal stable K562 cell lines expressing GFP-HO-1 all show the typical ER localization of GFP-fused wild type HO-1 (Figure 2B; cell lines 1-6). Notably, the nucleus is free of GFP-HO-1. In contrast, monoclonal stable K562 cell lines expressing GFP-tagged anchorless HO-1 all show cytosolic and nuclear localization (Figure 2C; cell lines 7-13) similar to translocated GFP-HO-1 [9].

### ER anchored HO-1 translocates under hypoxia in monoclonal stably transfected K562 cells

**Figure 3:**
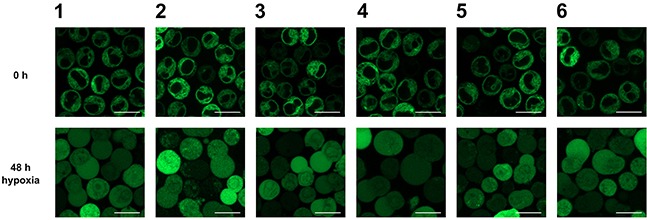
Analysis of the subcellular localization of GFP-HO-1 in the positively characterized stable K562 cell lines 1-6 after 48 h hypoxia (1 % O_2_) by confocal laser scanning microscopy Under normoxia GFP-HO-1 is localized at the ER membrane. The nucleus is free from GFP-HO-1. After 48 h incubation under hypoxia GFP-HO-1 translocated to cytosol and nucleus. Figure shows representative data from five pictures per sample out of three independent experiments on a CLSM at 37°C. Bar represents 20 μm.

HO-1 can be cleaved under stress conditions and subsequently translocates to the cytosol and nucleus [8–11]. To investigate whether this specific behavior of ER anchored HO-1 is also observed in our K562 cell model, we incubated our monoclonal stably transfected cells for 48 h under hypoxic conditions (1 % O_2_). We observed translocation of ER anchored HO-1 to the cytosol and nucleus in all six cell lines (Figure 3). After incubation under hypoxia, cell lines 1-6 showed the same distribution of the GFP fused HO-1 as cell lines 7-13, which mimic translocated HO-1. This shows that our GFP-HO-1 variant is able to translocate in K562 cells and that translocated GFP-HO-1 has the same phenotype as our anchorless model construct GFP-HO-1-ΔC266.

### ER anchored HO-1 catabolizes heme more efficiently than the anchorless HO-1 variant

**Figure 4:**
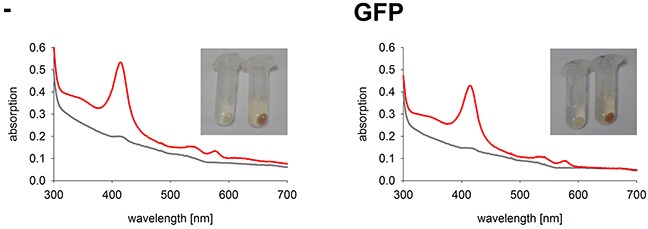
Hemoglobin synthesis experiments in a K562 cell line expressing GFP Cell pellets and hemoglobin absorbance spectra of a K562 cell line expressing GFP in comparison to untransfected K562. Cells were incubated for 5 days at 37°C and 5 % CO_2_ in K562 media. Grey curve: no media supplement. Red curve: supplement of 40 μM hemin. -: Untransfected K562. GFP: K562 expressing GFP. Cell pellets and spectra from one representative measurement are shown.

In comparison to other commonly used cell lines, K562 tolerate high hemin levels. When incubated with hemin K562 cells start to synthesize hemoglobin. With increasing synthesis of hemoglobin in K562 cells, a change in color from white to red is observed. In cell extracts the typical spectrum of hemoglobin with the Soret band at 415 nm and the two smaller α- and β-bands at 542 nm and 577 nm can be measured by UV-Vis spectrophotometry [24] (Figure 4). We used this property of K562 to draw conclusions about the activity of the GFP-fused HO-1 variants. When transfected active HO-1 catabolizes heme, less hemin is left for the formation of hemoglobin in comparison to untransfected cells. This should result in a smaller Soret peak of hemoglobin in the absorbance spectra and a shift of the color of the cell pellet from red to white.

**Figure 5:**
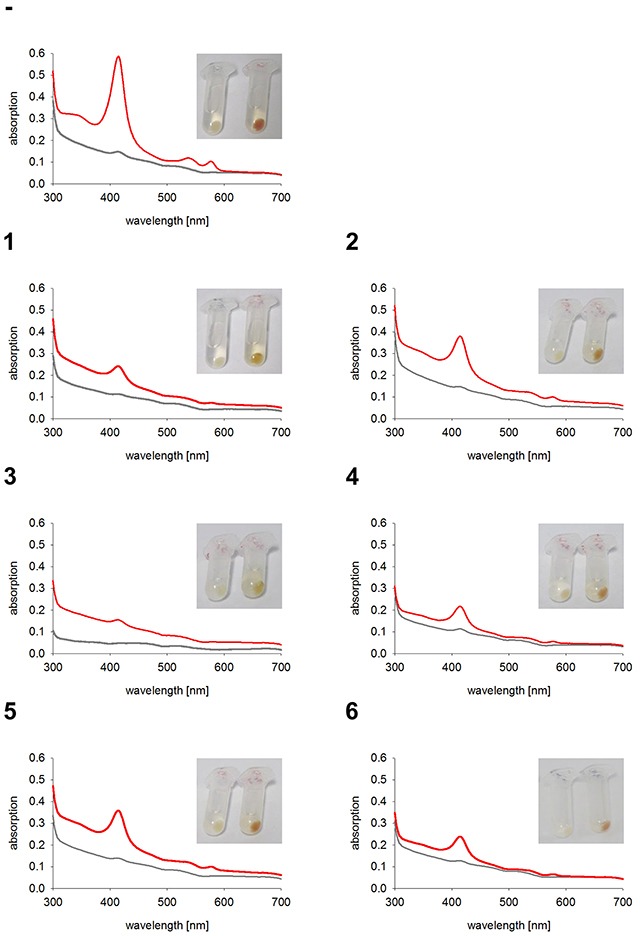
Hemoglobin synthesis experiments in K562 cell lines expressing ER anchored HO-1 Cell pellets and hemoglobin absorbance spectra of different K562 cell lines expressing the ER anchored HO-1 variant GFP-HO-1. Cells were incubated for 5 days at 37°C and 5 % CO_2_ in K562 media. Grey curve: no media supplement. Red curve: supplement of 40 μM hemin. -: Untransfected K562. 1-6: K562 expressing GFP-HO-1. Cell pellets and spectra from one representative measurement are shown.

**Figure 6:**
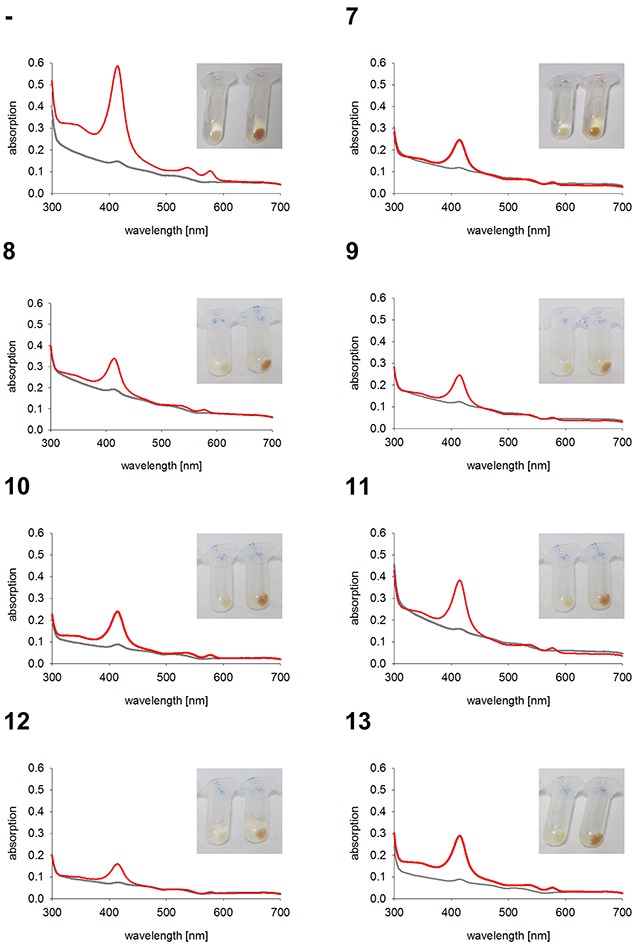
Hemoglobin synthesis experiments in K562 cell lines expressing anchorless HO-1 Cell pellets and hemoglobin absorbance spectra of different K562 cell lines expressing the anchorless HO-1 variant GFP-HO-1-ΔC266. Cells were incubated for 5 days at 37°C and 5 % CO_2_ in K562 media. Grey curve: no media supplement. Red curve: supplement of 40 μM hemin. -: Untransfected K562. 7-13: K562 expressing GFP-HO-1-ΔC266. Cell pellets and spectra from one representative measurement are shown.

As expected, the two control cell lines, untransfected K562 and GFP expressing K562, both have red colored cell pellets and similar hemoglobin spectra (Figure 4). All monoclonal stable K562 cell lines with the construct GFP-HO-1 show less colored cell pellets than untransfected K562 and a decrease in the Soret band measured by UV-Vis spectroscopy (Figure 5; cell lines 1-6). Cell lines expressing the anchorless variant also have whiter cell pellets and a smaller Soret band than untransfected K562 (Figure 6; cell lines 7-13). This indicates that both types of HO-1 constructs are active as a portion of hemin is catabolized and not available for hemoglobin formation. Based on inspection of cell pellets, cell lines expressing the anchorless HO-1 construct seem to have weaker HO-1 activity in comparison to cell lines expressing the full length ER anchored HO-1.

**Figure 7:**
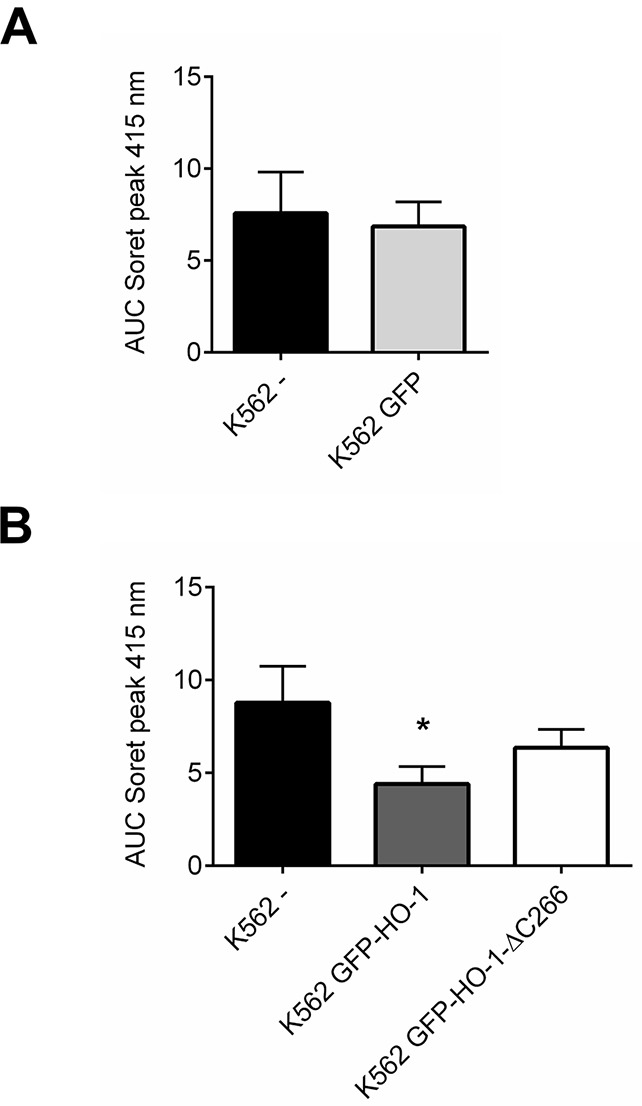
Comparison between HO-activity of untransfected or GFP expressing control cells and K562 cell lines expressing ER resident HO-1 or the anchorless HO-1 variant AUCs of the Soret peak at 415 nm were calculated for each cell line. **(A)** Data from four independent experiments were averaged per group to compare the HO-activity in untransfected K562 with K562 expressing GFP. **(B)** Data from four independent experiments were averaged per group to compare the HO-activity in untransfected K562 with the HO-activity in cell lines expressing ER anchored GFP-HO-1 and anchorless GFP-HO-1-ΔC266. Error bars show the SEM. * significant compared to untransfected K562 (p<0.05).

To quantify the reduction of hemoglobin synthesis upon HO-1 expression, we calculated the area under the curve (AUC) of the Soret peak at 415 nm. As expected untransfected K562 and K562 GFP synthesized similar amounts of hemoglobin (Figure 7A), because both lack overexpressed HO-1. Data of cell lines expressing the same construct were averaged to compare the activity of K562 cells expressing ER resident HO-1 or anchorless HO-1 with untransfected K562 cells (Figure 7B). Both constructs lead to a decrease in hemoglobin formation due to hemin catabolism by HO-1, when compared with untransfected K562. However, ER anchored HO-1 had higher activity than the anchorless variant compared to untransfected K562 cells. This is shown by the significantly lower AUC value of the Soret peak, while the group with the nuclear and cytosolic HO-1 just shows a moderate decrease that does not reach statistical significance.

### K562 cell lines expressing ER anchored full length HO-1 show sensitivity to imatinib

**Figure 8:**
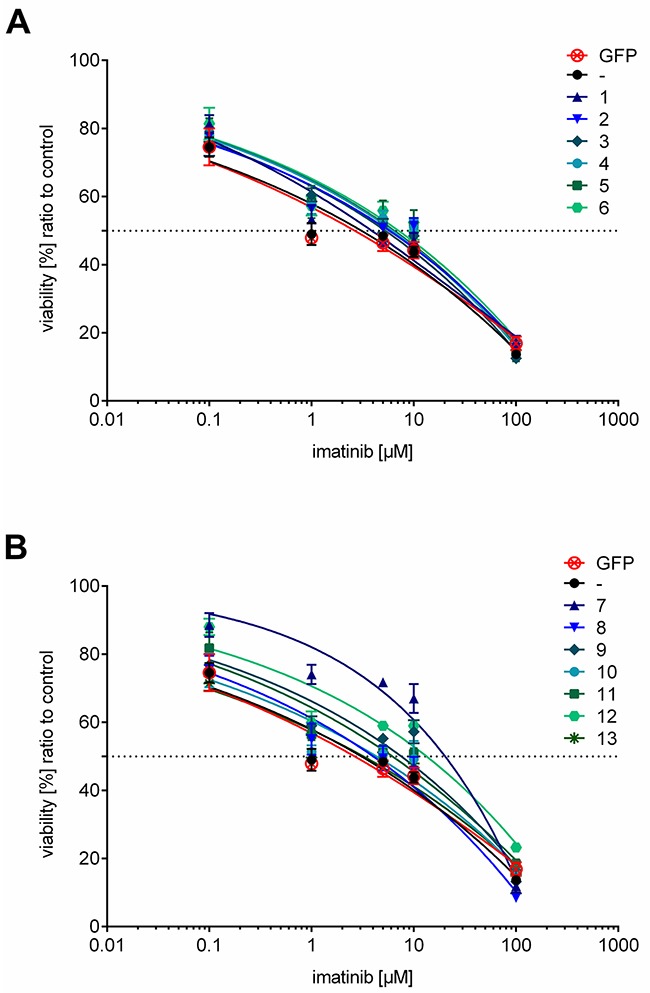
Dose-response curves of cell lines expressing ER resident or anchorless HO-1 treated with imatinib **(A)** Dose-response curves for cell lines expressing ER anchored GFP-HO-1. **(B)** Dose-response curves for cell lines expressing the anchorless HO-1 variant GFP-HO-1-ΔC266. Dose-response curves of untransfected K562 and K562 GFP are added to both graphs for comparison. The curves show data from triplicate measurements of at least three independent experiments and the calculated dose-response curves for each cell line. Error bars show the SEM.

To analyze the effect of ER resident overexpressed HO-1 in K562 cells on imatinib resistance we measured cell viability with an MTT based cell viability assay of all monoclonal stable K562 cell lines and untransfected K562 or K562 GFP as controls. Dose-response curves of K562 cells expressing the ER anchored HO-1 variant were generated (Figure 8A) and the corresponding IC50 values are given in Table 1 (IC50, mean ± SEM). The IC50 values of cell lines expressing ER anchored HO-1 lie between 3.78 ± 1.33 μM and 7.08 ± 1.35 μM and are well within the range of the IC50 value of untransfected cells (2.87 ± 1.41 μM) or K562 GFP (2.35 ± 1.38 μM). Taken together this indicates that all cell lines with ER anchored full length HO-1 show sensitivity to imatinib treatment just like untransfected K562 cells or K562 GFP.

**Table 1:** IC50 values of the tested monoclonal stable K562 cell lines and untransfected K562

Cell line	IC50[μM]	SEM IC50[μM]	IC50[ng/ml]	SEM IC50[ng/ml]
K562 GFP	2.35	1.38	1386	814
K562 -	2.87	1.41	1692	831
K562 GFP-HO-1 1	3.78	1.33	2229	784
K562 GFP-HO-1 2	5.68	1.22	3349	719
K562 GFP-HO-1 3	5.19	1.23	3061	725
K562 GFP-HO-1 4	6.07	1.24	3579	731
K562 GFP-HO-1 5	5.99	1.27	3532	749
K562 GFP-HO-1 6	7.08	1.35	4175	796
K562 GFP-HO-1-ΔC266 7	19.76 *	1.19	11652 *	702
K562 GFP-HO-1-ΔC266 8	3.72	1.30	2193	767
K562 GFP-HO-1-ΔC266 9	7.57	1.26	4464	743
K562 GFP-HO-1-ΔC266 10	3.96	1.54	2335	908
K562 GFP-HO-1-ΔC266 11	5.65	1.41	3332	831
K562 GFP-HO-1-ΔC266 12	12.35 *	1.57	7283 *	926
K562 GFP-HO-1-ΔC266 13	2.92	1.30	1722	767

The first column shows the name of the tested cell line, the second and fourth column the determined IC50 value as mean of at least three independent experiments in μM or ng/ml, the third and fifth column the SEM of the IC50 in μM or ng/ml.

* significant increase of IC50 compared to untransfected K562 or K562 GFP (p<0.05).

### K562 cell lines expressing anchorless HO-1 may develop resistance against imatinib

To evaluate the role of the anchorless HO-1 in imatinib resistance, we measured dose-response curves (Figure 8B) and determined IC50 values (Table 1). Five cell lines (8, 9, 10, 11 and 13) showed IC50 values from 2.92 ± 1.30 μM to 7.57 ± 1.26 μM that were again similar to the IC50 value of untransfected cells or K562 GFP. However, two cell lines (7 and 12) showed significantly higher IC50 values: 19.76 ± 1.19 μM and 12.35 ± 1.57 μM, respectively. Hence, cell lines expressing anchorless HO-1 can be divided into two groups: imatinib sensitive and imatinib resistant cell lines.

### Cell morphology confirms imatinib sensitivity or resistance of selected K562 cell lines

**Figure 9:**
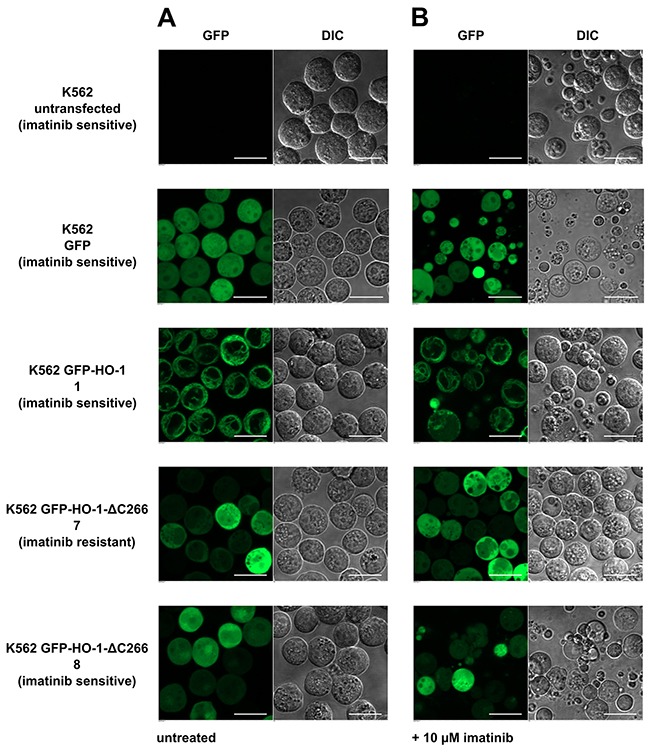
Confocal laser scanning microscopy of selected imatinib treated K562 cell lines **(A)** Untreated. **(B)** 24 hours after incubation with 10 μM imatinib. Figure shows representative data from five pictures per sample out of three independent experiments on a CLSM at 37°C. DIC: differential interference contrast. Bar represents 20 μm.

To validate these findings, one representative cell line from each group was selected for confocal laser scanning microscopy. Cells were imaged untreated and after treatment with 10 μM imatinib for 24 h. The imatinib concentration of 10 μM corresponds to a plasma concentration of 5897 ng / ml. Trough plasma concentrations of imatinib are in the range of 1000 ng / ml [25]. The highest reported peak plasma concentration (C_max_) of imatinib in CML patients was 4478 ng / ml in a study on pharmacokinetics of imatinib [26]. The chosen concentration of 5897 ng / ml therefore would probably be reached only with exceptionally high doses in patients and thus represents a good test dose for imatinib resistance. No change in morphology was observed in the imatinib resistant K562 cell line 7 expressing anchorless HO-1 (Figure 9). In contrast, all other tested cell lines showed morphological signs of apoptosis which is seen best in the differential interference contrast (Figure 9, DIC). This indicates that anchorless HO-1 can promote imatinib resistance in concert with other factors that is likely clinically relevant.

## DISCUSSION

An altered expression of HO-1 in cancer and leukemic cells was observed in several studies [2–4, 27]. In addition, HO-1 translocation from the ER to the cytosol and nucleus has been reported [3, 11–14]. Whether HO-1 represents just a marker of neoplastic disease, or whether there is a causal relationship between HO-1 and malignant growth is unclear until now. However, it is this distinction that decides whether HO-1 is indeed a promising novel target of cancer therapy [1]. Translocation of HO-1 to the cytosol and nucleus has been explicitly linked to imatinib resistance in the chronic myelogenous leukemia cell line K562 [14]. To challenge these findings, we decided to study whether imatinib resistance can be induced by overexpression of HO-1 variants. Our results show that overexpression of full length ER resident HO-1 has no effect on imatinib resistance. However, overexpression of an HO-1 variant mimicking translocated HO-1 (anchorless HO-1) resulted in imatinib resistance in two out of seven cell lines. This is in full agreement with the findings of Tibullo et al. showing that the translocated, anchorless HO-1 causes imatinib resistance, rather than the ER resident form [14].

The observation that only two out of seven cell lines were resistant clearly shows that anchorless HO-1 is not a direct cause of imatinib resistance. However, it is in fact a high percentage of resistant cells from the clinical perspective as CML patients have to take imatinib for a long period. To develop a clinically relevant imatinib resistance one single resistant cell is enough, because imatinib sensitive leukemia cells are killed regularly and imatinib resistant leukemia cells survive and accumulate over time [20, 28]. This is a well-known principle in long term hematological-oncological therapy with tyrosine kinase inhibitors [29]. For example in a study with 85 CLL (chronic lymphocytic leukemia) patients treated with the Bruton's tyrosine kinase (BTK) inhibitor ibrutinib 7 patients developed Richter`s transformation, a more aggressive lymphoma form, during ibrutinib treatment [30].

Our findings show that overexpression of anchorless HO-1 alone is not sufficient to cause imatinib resistance. A possible explanation is that translocated HO-1 has an effect on an intervening factor that causes imatinib resistance with some variability. This idea would fit to more recent work by Tibullo et al. who showed that nuclear HO-1 promotes genomic instability in malignant cells [13]. This is also in line with signs of imatinib resistance occurring as a consequence of somatic mutations in some but not all cell lines. Nuclear HO-1 has been shown to interact with Nrf2 (NF-E2 related factor 2) [31] and additional transcription factors such as STAT3, CDP, Brn-3, CBF and AP-2 [8]. Thus, a direct or indirect influence on DNA repair mechanisms is well conceivable [13].

It cannot be ruled out completely that inactivation of unrelated genes due to genomic integration of constructs has an influence on imatinib resistance. Nevertheless, it is unlikely that coding genes are inactivated, it is very unlikely that these coding genes have an influence on imatinib resistance and it is extremely unlikely that this happens twice and only in cell lines overexpressing the anchorless construct GFP-HO-1-ΔC266 but not in cell lines expressing GFP-HO-1 or GFP.

Our finding that overexpression of ER resident HO-1 does not lead to imatinib resistance, argues against the idea that products of the enzymatic heme oxygenase reaction such as biliverdin / bilirubin or the gaseous molecule carbon monoxide induce imatinib resistance. It is rather likely that mutations in other signal pathways of the leukemic cell together with imatinib treatment, lead to upregulation of HO-1 in the leukemic cell. This might allow the cell to cope with imatinib toxicity and the metabolic derangements typical of malignant cells [32].

HO-1 enables renal carcinoma cells with a defect in fumarate hydratase to proliferate despite the defect in the tricarboxylic acid cycle [27]. In this study, HO-1 was said to be synthetically lethal with the tumor suppressor fumarate hydratase [27]. Two genes are synthetically lethal if mutation of either alone is compatible with viability but mutation of both leads to death [33]. This represents a unique opportunity because targeting a gene that is synthetically lethal to a cancer-relevant mutation should kill only cancer cells and spare normal cells [33]. In analogy to a cancer relevant mutation (such as the BCR-ABL oncogene) that leads to a compensating HO-1 increase, imatinib treatment can also lead to a similar compensating HO-1 increase. In both cases, the compensating gene represents a promising drug target or co-target. This idea is supported by the finding that inhibition of HO-1 induces apoptosis in BCR-ABL-positive B-ALL (acute lympohoblastic leukemia) and increases the sensitivity to treatment by imatinib [34]. Similarly, HO-1 has recently been found to be upregulated in pancreatic cancer treated with imatinib [35] and was also suggested as co-target of imatinib treatment.

It has previously been shown that anchorless HO-1 is less active than ER anchored HO-1 [8]. We can confirm this with our hemoglobin synthesis experiments. In cell lines expressing anchorless HO-1 more hemoglobin could be synthesized than in cell lines expressing ER anchored HO-1. Hence, ER resident GFP-HO-1 had higher heme catabolizing activity than the anchorless variant GFP-HO-1-ΔC266. The reason for this lies in the subcellular localization of HO-1. CPR is the electron donor for heme catabolism [36] and is located at the ER membrane. Although anchorless HO-1 still contains the CPR and BVR (biliverdin reductase) binding sites [37, 38], it is less likely to form a complex with CPR as HO-1's ER anchor increases CPR binding affinity [39, 40]. Interaction of CPR with anchorless HO-1 is still possible in the cytosol, but very unlikely in the nucleus. However, there may be alternative electron donors in the nucleus which may provide electrons to translocated HO-1, since it has been shown that modulation of some transcription factors by nuclear HO-1 depend on heme degrading activity [8].

Heme induced hemoglobin synthesis in K562 cells also reflects erythroid differentiation of the megakaryocyte-erythroid bone marrow progenitor cell [24, 41]. It has been shown that erythropoietin protects leukemia cells from imatinib-induced killing by promoting erythroid differentiation [42]. This is in line with the effect of heme which promotes both imatinib resistance and erythroid differentiation [28]. Results from Jacquel et al. indicate that imatinib treatment of K562 cells either leads to apoptosis or erythroid differentiation [43]. Taken together erythroid differentiation may play a role in HO-1 mediated drug resistance, although our experiments do not indicate any correlation between hemoglobin formation from supplemented hemin and imatinib resistance.

In summary, we suggest that HO-1 plays a dual role in imatinib resistant CML: endogenous ER resident HO-1 can represent a survival factor for leukemic cells, but overexpression of ER resident HO-1 does not lead to imatinib resistance, whereas translocation of HO-1 to the nucleus and cytosol supports imatinib resistance in some but not all cell lines. We conclude that further studies should focus on the potential of inhibiting the translocation mechanism of HO-1. This might be achieved by the avoidance of hypoxia and the use of SPP inhibitors and may represent a general strategy for the prevention of genomic instability and drug resistance of cancer cells.

## MATERIALS AND METHODS

### Materials

Unless stated otherwise, chemicals were purchased in high purified quality from Sigma-Aldrich Chemie GmbH (Steinheim, Germany), Applichem GmbH (Darmstadt, Germany) or Serva Electrophoresis GmbH (Heidelberg, Germany). Cell culture media were received from Life Technologies GmbH, Invitrogen™ (Darmstadt, Germany) or BIO & Sell e. K. (Feucht, Germany).

### Cloning of HO-constructs

GFP-HO-1 and the anchorless construct GFP-HO-1-ΔC266 were cloned as described before [9] corresponding to the UniProt accession code P09601.

### Cell culture

K562 were purchased from DSMZ (Leibniz Institute DSMZ, German Collection of Microorganisms and Cell Cultures, Braunschweig, Germany, DSMZ-No. ACC 10) [41]. They were cultivated in RPMI (Roswell Park Memorial Institute) 1640 with 10 % fetal bovine serum and 1 % Penicillin/Streptomycin at 37°C with 5 % CO_2_. For cultivation of stable K562 cell lines 1 % G418 was added.

### Generation and characterization of monoclonal stable K562 cell lines

Stable K562 cell lines were created as described in Zeyda et al. [44]. Transient transfection of K562 cells was made by electroporation with Neon^®^ Transfection System (Thermo Fisher Scientific, Walthram, USA) according to manufacturer's recommendations. Freshly electroporated cells were kept in media without antibiotics. 48 h after transient transfection cells were sorted for the first time. GFP+ cells were kept and cultivated in RPMI 1640 with 10 % fetal bovine serum and 1 % Penicillin/Streptomycin + 1 % G418. Sorting of GFP+ cells in a batch was done for two more times, each after 10-12 more days of cultivation. After a minimum of three batch sorts, single GFP+ cells were seeded into 96 well plates for achieving monoclonal stable cell lines. Cell sorting was carried out on a BD FACS Aria II (Becton Dickinson GmbH, Heidelberg, Germany) at the platform for flow cytometry of the Helmholtz Center for Infection Research, Braunschweig, Germany. All monoclonal stable K562 cell lines were screened for GFP and HO-1 expression by Western blot analysis with specific anti-GFP and anti-HO-1 antibodies and correct localization of the HO-1 variant using live cell imaging. In cell lines expressing ER anchored HO-1 hypoxia mediated translocation was additionally tested. Just positively characterized cells were included in further experiments.

### Cell extracts

Cell extracts were made by sonification of K562 cells (Sonoplus HD 2070, Bandelin electronic GmbH & Co. KG, Berlin, Germany) in TEA (triethanolamine)-lysis-buffer (50 mM TEA, 1 mM EDTA, pH=7.4) containing one tablet cOmplete protease inhibitor cocktail per 50 ml (Roche Diagnostics Deutschland GmbH, Mannheim, Germany) and subsequent centrifugation for 30 min at 21 000 × g and 4°C. Overall protein concentration of the cell extracts were determined by Bradford assay [45].

### SDS-PAGE and western blot

For Western blot analysis cell extracts containing 90 μg protein and the equivalent volume of sodium dodecyl sulphate (SDS) sample-buffer (1 % SDS, 100 mM DTT (Dithiotreitol), 50 mM Tris, 30 % Glycerol, pH=7.5) were used. The samples were cooked for 3 min at 99°C. Afterwards bromophenol blue was added and the samples were loaded on 10 % gels. PageRuler™ Prestained Protein Ladder and PageRuler™ Unstained Protein Ladder (Thermo Scientific, Walthram, USA) were used for size control. After SDS-PAGE gels were blotted on nitrocellulose membranes, stained with Ponceau S and blocked for at least 1 hour in TBST (Tris-buffered saline with Tween^®^20) buffer (10 mM Tris–HCl, 150 mM NaCl, 0.1 % Tween^®^ 20, pH=8.0) containing 5 % non-fat dry milk. For detection we used used rabbit-anti-HO-1 (1:5000, Stressgen, Enzo Life Sciences, Lörrach, Germany), rabbit-anti-GFP (1:2000, Clontech Laboratories, Inc., Mountain View, USA) and mouse-anti-actin (1:2000, Sigma-Aldrich Chemie GmbH, Steinheim, Germany). As secondary antibody we used horseradish peroxidase-conjugated anti-rabbit or anti-mouse IgGs (1:2000, Cell Signaling Technology, Inc., Danvers, USA). HO-1 antibody was diluted in TBST buffer, containing 1 % non-fat dry milk, all other antibodies were diluted in TBST buffer. The membranes were incubated for 1-2 h at room temperature with primary antibodies, washed three times for 5 minutes with TBST and then incubated with the secondary antibodies for 45 min. After another three washing steps the membranes were detected with Lumi-Light^PLUS^ Western Blotting Substrate (Roche Diagnostics Deutschland GmbH, Mannheim, Germany) according to manufacturer`s recommendations in an ECL ChemiLux Imager (Intas, Göttingen, Germany).

### Live cell imaging

Stable transfected K562 cell lines were imaged at 37°C on a Nikon Ti-E microscope equipped with an incubation chamber (Okolab) using a 60 × oil immersion objective (NA 1.4, Nikon). A focused 488 nm laser was used for GFP excitation. Emission was measured between 500-550 nm. For translocation experiments all cell lines expressing GFP-HO-1 were imaged before and after incubation under hypoxia (1 % O_2_) for 48 h. For cell viability experiments selected cell lines were incubated with 10 μM imatinib for 24 h, imaged and compared to cells without imatinib stimulation. For data collection and picture editing we used NIS-Elements Ar Microscope Imaging Software (Nikon Instruments Europe BV, Amsterdam, Netherlands).

### Hemoglobin formation experiments

1 Million cells per sample of untransfected K562 and stable transfected K562 cell lines were cultivated at 37°C and 5 % CO_2_ in RPMI 1640 with 10 % fetal bovine serum and 1 % Penicillin/Streptomycin once with, once without the supplement of 40 μM hemin. Five days later the cells were centrifuged at 900 × g, 4°C for 5 min, the supernatant was refused and the pellet was washed in 1 ml PBS (phosphate-buffered saline). After washing cells were centrifuged at 2000 × g, 4°C for 5 min and the pellet was photographed. After removing the supernatant cells were resuspended in 500 μl fresh PBS, sonified as described above and centrifuged at 15000 × g, 4°C for 30 min. Absorbance spectra of the supernatants were measured on a UV-Vis spectrophotometer Cary 50 (Varian/Agilent Technologies, Waldbronn, Germany).

### Cell viability assay

20000 cells per well were seeded in RPMI 1640 with 10 % fetal bovine serum and 1 % Penicillin/Streptomycin in 96 well plates. The next day they were stimulated with increasing concentrations of imatinib (0/0.1/1/5/10/100 μM) and cultivated for 22 more hours, before MTT (3-(4,5-Dimethylthiazol-2-yl)-2,5-diphenyltetrazolium-bromide) was added. After incubation for 2 more hours cells were harvested and resuspended in extraction buffer (DMF (dimethylformamide) / H_2_O (*v/v*) 1:1, 10 % SDS (*w/v*)). Cell viability was measured colorimetric at 550 nm on a Sunrise™ Absorbance Reader (Tecan Deutschland GmbH, Crailsheim, Germany). Data was calculated as ratio to untreated control.

### Statistical analysis

Data values of at least three independent experiments were analyzed by one-way ANOVA (analysis of variance) followed by paired student`s t-test versus untransfected K562 or K562 GFP. P-values <0.05 (*) were considered significant. Data are presented with indicated error bars showing ± SEM.

## Abbreviations

ALL: acute lymphoblastic leukemia
ANOVA: analysis of variance
AP-2: activator protein-2
AUC: area under the curve
Brn-3: group of related transcription factors in the POU family
BTK: Bruton's tyrosine kinase
BVR: biliverdin reductase
CBF: core-binding factor
CDP: CCAT displacement protein
CLSM: confocal laser scanning microscope
C_max_: maximum concentration
CLL: chronic lymphocytic leukemia
CML: chronic myelogenous leukemia
CPR: cytochrome P450 reductase
DIC: differential interference contrast
DMF: dimethylformamide
DNA: deoxyribonucleic acid
DTT: dithiothreitol
EDTA: ethylenediaminetetraacetic acid
ER: endoplasmic reticulum
GFP: green fluorescent protein
HO-1: heme oxygenase-1
IC50: half maximal inhibitory concentration
K562: cell line from chronic myeloid leukemia in blast crisis
MTT: 3-(4,5-Dimethylthiazol-2-yl)-2,5-diphenyltetrazolium-bromide
PBS: phosphate-buffered saline
Nrf2: NF-E2 related factor 2
RPMI: Roswell Park Memorial Institute
SDS: sodium dodecyl sulphate
Sf-9: ovary cell line from Spodoptera frugiperda
SPP: signal peptide peptidase
STAT3: signal transducer and activator of transcription 3
TBST: Tris-buffered saline with Tween^®^20
TEA: triethanolamine
UV-Vis: ultraviolet-visible
ZnPP: zinc protoporphyrine.
